# Melatonin ameliorates H_2_O_2_-induced oxidative stress through modulation of Erk/Akt/NFkB pathway

**DOI:** 10.1186/s40659-018-0168-5

**Published:** 2018-06-11

**Authors:** Mahammed Moniruzzaman, Indranath Ghosal, Debjit Das, Suman Bhusan Chakraborty

**Affiliations:** Fish Endocrinology Research Unit, Department of Zoology, University of Calcutta, 35 Ballygunge Circular Road, Kolkata, West Bengal 700019 India

**Keywords:** Fish, Melatonin, Hepatocytes, Heat shock proteins, Oxidative stress

## Abstract

**Background:**

Improper control on reactive oxygen species (ROS) elimination process and formation of free radicals causes tissue dysfunction. Pineal hormone melatonin is considered a potent regulator of such oxidative damage in different vertebrates. Aim of the current communication is to evaluate the levels of oxidative stress and ROS induced damage, and amelioration of oxidative status through melatonin induced activation of signaling pathways. Hepatocytes were isolated from adult *Labeo rohita* and exposed to H_2_O_2_ at three different doses (12.5, 25 and 50 µM) to observe peroxide induced damage in fish hepatocytes. Melatonin (25, 50 and 100 μg/ml) was administered against the highest dose of H_2_O_2_. Enzymatic and non-enzymatic antioxidants such as malondialdehyde (MDA), superoxide dismutase (SOD), catalase (CAT) and glutathione (GSH) was measured spectrophotometrically. Expression level of heat shock proteins (HSP70 and HSP90), HSPs-associated signaling molecules (Akt, ERK, cytosolic and nuclear NFkB), and melatonin receptor was also measured by western blotting analysis.

**Results:**

H_2_O_2_ induced oxidative stress significantly altered (P < 0.05) MDA and GSH level, SOD and CAT activity, and up regulated HSP70 and HSP90 expression in carp hepatocytes. Signaling proteins exhibited differential modulation as revealed from their expression patterns in H_2_O_2_-exposed fish hepatocytes, in comparison with control hepatocytes. Melatonin treatment of H_2_O_2_-stressed fish hepatocytes restored basal cellular oxidative status in a dose dependent manner. Melatonin was observed to be inducer of signaling process by modulation of signaling molecules and melatonin receptor.

**Conclusions:**

The results suggest that exogenous melatonin at the concentration of 100 µg/ml is required to improve oxidative status of the H_2_O_2_-stressed fish hepatocytes. In H_2_O_2_ exposed hepatocytes, melatonin modulates expression of HSP70 and HSP90 that enable the hepatocytes to become stress tolerant and survive by altering the actions of ERK, Akt, cytosolic and nuclear NFkB in the signal transduction pathways. Study also confirms that melatonin could act through melatonin receptor coupled to ERK/Akt signaling pathways. This understanding of the mechanism by which melatonin regulates oxidative status in the stressed hepatocytes may initiate the development of novel strategies for hepatic disease therapy in future.

## Background

Exposure to contaminated environment may cause different biochemical and physiological alteration at tissue and cellular levels. Physiological changes are often regulated by variations in the neuro-endocrine system. In addition to neuro-endocrine stress response in the organismal levels, there occurs a cellular stress response following exposure to different toxic substances. Different such stressors enhance the levels of free radicals and cause oxidative damages through generation of reactive oxygen species (ROS) [1]. ROS is also generated naturally in all aerobic organisms. Improper control on ROS elimination process and formation of free radicals causes tissue dysfunction and altered physiology. Hydrogen peroxide (H_2_O_2_) is a well-known ROS having potential to induce liver disorders [2].

Pineal hormone melatonin (*N*-acetyl-5-methoxytryptamine) is vital due to its neuroendocrine function and universal occurrence throughout different taxa including unicellular eukaryotes, invertebrates and vertebrates [3]. Melatonin is considered a potent candidate in regulation of oxidative damage in different vertebrates including fish [1]. Circulating profiles of this hormone exhibit a close relation with the development of antioxidant status of different tissues. Melatonin plays vital role in antioxidant activity by converting upon oxidation to a number of antioxidant compounds that includes cyclic 3-hydroxymelatonin, *N*1-acetyl-*N*2-formyl-5-methoxykynuramine, and *N*1-acetyl-5-methoxykynuramine. Because of such array of compounds, melatonin is considered to be a wide range antioxidant and potentially much powerful than glutathione in neutralizing free radicals and more effective than other frequent antioxidants in protecting cellular membranes [2]. It stimulates synthesis of different antioxidative enzymes such as superoxide dismutase, glutathione peroxidase, glutathione reductase and catalase. It is also a potent antioxidant to scavenge different free radicals and thereby inhibits lipid peroxidation. It has been reported that endogenously synthesized or exogenously added melatonin may achieve its potent remedial function against stress related disease by modulating different transcription factors though its receptor protein [4]. Therefore, investigation on melatonin receptor expression may be a key point to evaluate the potential mechanism of melatonin action. Report also claimed that melatonin enhances intracellular glutathione levels by stimulating the key regulatory enzyme, γ-glutamyl cysteine synthase [5]. Recently melatonin has been applied to treat liver dysfunction to reduce oxidative stress [6]. However, no such documentation regarding the hepatoprotective efficacy of melatonin was done in fish at cellular or tissue levels till date.

Heat-shock proteins (HSPs) play a vital role in the cell and tissue physiology. HSPs maintain translational regulation through their molecular chaperone function that allows them to recognize damaged proteins. HSPs lead such proteins into repair and proper folding pathways or to proteosomal proteolysis. Heat shock protein 70 (HSP70) may act as biomarker, particularly after ROS toxicity in fish. The enhanced levels of HSP70 accumulation in stressed cells shields the destruction of protein machinery and associated functions [7]. Earlier studies also recorded 90-kDa heat shock protein, HSP90, as a crucial molecular chaperone participating in the cyto-protection of eukaryotic cells during stress [8]. Cells have developed sophisticated mechanisms associated with induction of HSPs to maintain cellular homeostasis and try to cope with the overloading of reactive oxygen species (ROS) produced during oxidative stress. An earlier study also revealed that melatonin is the key regulatory molecule to modulate the HSP activity during stress recovery [9].

It has been shown that ERK is activated by accumulation of hydrogen peroxide (H_2_O_2_), the profuse reactive oxygen metabolite of cell [10]. Cells respond to oxidative damage through the commencement of multiple signal transduction pathways that possibly coordinate the diverse cellular responses. ERK cascade is significant signaling pathway that plays major roles in the regulation of intracellular metabolism and serial expression of several genes. It has a critical role in the adaptive responses to thermal, osmotic and other stresses that causes oxidative injury [11].

Nuclear factor-kappa binding (NFkB) is a protein complex that controls transcription of numerous genes. It is involved in cellular response to stimuli such as stress, free radicals, ROS and cytokines. Under non-stressed condition, this protein resides in cytoplasm, associated with an inhibitory factor (IκB). NFkB activation in response to a stimulus occurs as a result of phosphorylation and proteolytic degradation of IκB. This unveils the nuclear localization signal of NFkB and its translocation into the nucleus to regulate the gene expression.

Previous studies reported that Akt signaling pathway regulates the expression of Hsp70, which critically contributes to Hsp90-chaperone function [12]. HSP is also found to be critical to modulate the NFkB signaling pathway towards tolerance of oxidative stress [13]. In some earlier reports melatonin was noted to induce stem cell differentiation through formation of MT/MEK/ERK1/2 complexes, causing expression of different genes [14, 15]. Consistent with all these earlier studies, current study has been undertaken to unmask the consequences of H_2_O_2_-induced oxidative stress in isolated fish hepatocytes and how much the acclimatization process and defense strategy against such stress can be influenced by exogenous administration of melatonin. Therefore outcome of the current work will be helpful to find out the significance of melatonin in removal of superoxide radical generated hepatocyte stress through cross-talk between antioxidant status, molecular chaperon and transcription factor NFkB. The present study is also the first attempt to demonstrate the mechanism of exogenous melatonin to combat H_2_O_2_-induced free radical damage in the fish hepatocytes and its consequences on the signaling cascade by focusing the role of different antioxidative agents. We also intend to unveil the possible mechanism that involves the melatonin induced activation of ERK via the up regulation MT1 receptor protein in the fish hepatocytes.

## Methods

### Hepatocyte isolation, culture and experimental set up

Adult *Labeo rohita* were used for isolation of hepatocytes. The investigation conforms with the Guide for the Care and Use of Laboratory Animals published by the US National Institute of Health (NIH Publication No. 85-23, revised 1996) and was also approved by the Institutional Animal Ethics Committee, University of Calcutta (Registration #885/ac/05/CPCSEA), registered under the “Committee for the Purpose of Control and Supervision of Experiments on Laboratory Animals” (CPCSEA), Ministry of Environment and Forests, Government of India. Fishes were anaesthetized and liver was perfused via portal vein by using modified Hanks medium. Perfused liver was isolated, minced and digested with type IV collagenase in original Hanks medium for 1 h at 37 °C. Digested cell suspension was filtered through two layers of nylon mesh and supernatant was removed. Cells were then resuspended in MEM supplemented with antibiotics (penicillin/streptomycin) and 0.2% BSA. Viability of the cells was determined using the trypan blue exclusion method. Cells were plated in collagen coated plates and cultured in a humidified 5% CO_2_ atmosphere at 37 °C.

Isolated hepatocytes were equally divided (minimum 10^6^ cells/well) into eight groups for undergoing following separate treatments: (a) control [Treatment group 1], (b) H_2_O_2_ (12.5 µM) [Treatment group 2], (c) H_2_O_2_ (25 µM) [Treatment group 3], (d) H_2_O_2_ (50 µM) [Treatment group 4], (e) H_2_O_2_ (50 µM) + melatonin (25 μg/ml) [Treatment group 5], (f) H_2_O_2_ (50 µM) + melatonin (50 μg/ml) [Treatment group 6], (g) H_2_O_2_ (50 µM) + melatonin (100 μg/ml) [Treatment group 7] and (h) melatonin (100 μg/ml) [Treatment group 8].

### Sub-cellular fractionation

The extraction procedure was performed as described by Baghirova et al. [16] with minor modifications. Ice cold lysis buffer supplemented with 5 µl protease inhibitor cocktail was added to the cell and disrupted the tissue for about 10 s using a hand held tissue homogenizer. Tissue suspension was then sonicated and centrifuged at 500*g* for 10 min at 4 °C to filter the homogenate. After discarding the trapped tissue particles the pellet was resuspended by gently pipetting up and down. 500 µl of ice cold lysis buffer supplemented with 5 µl of protease inhibitor cocktail was added. The homogenate was centrifuge at 4000*g* for 10 min at 4 °C. The supernatant was collected as the cytosolic proteins. The pellet was resuspended by gently pipetting up and down in ice cold lysis buffer supplemented with 10 µl of protease inhibitor cocktail. After 30 min of incubation homogenate was centrifuged at 8000*g* for 10 min at 4 °C. After discarding the supernatant 500 units of benzonase (Sigma, E1014) with 20 µl of water was added to the pellet and resuspension was done. 500 µl of ice cold lysis buffer with 5 µl of protease inhibitor cocktail was added to the benzonase digested pellet, re-sonicated with brief pulse for 30 s and incubated for 10 min. The insoluble material was centrifuged at 12,000–13,000*g* for 10 min at 4 °C and the supernatant was collected as nuclear protein fractions.

### Estimation of antioxidative parameters

Enzymatic and non-enzymatic antioxidants such as malondialdehyde (MDA), superoxide dismutase (SOD), catalase (CAT) and GSH (glutathione) was measured spectrophotometrically as described earlier [17]. Protein concentrations in the supernatants were determined using Bradford’s procedure.

### Protein immunoblot analysis

Protein expression was analyzed on 12.5% Laemmli SDS-PAGE and processed for immunoblot on PVDF membrane by wet electroblotting method. Primary antibody used (1:1000 dilution) against heat shock protein (HSP 70 and HSP90), Akt, Erk 1/2, NFkB (cytosolic and nuclear) followed by incubation with the respective secondary antibody (dilution 1:500). Individual band intensity of each immunoblot quantified by densitometry using ImageJ Software after normalizing with internal control (β-actin) [17].

### Determination of hydroxyl and peroxide scavenging activity

Ability of the melatonin to scavenge hydroxyl and peroxide radical was assessed by the standard method [18, 19] respectively.

### Statistical analysis

All the data were expressed as mean ± SE and analyzed by univariate multiway ANOVA, where Pr > F (at 5% significance level) values indicated significance. The population means were compared by a post hoc multiple comparison testing, taking *P* value < 0.05 as the threshold. Values of melatonin receptor in isolated hepatocytes were plotted against each of the signaling molecules [Akt, Erk 1/2 and NFkB (cytosolic and nuclear)] for linear regression analysis. R^2^ value of each of the analysis was calculated to understand the correlation.

## Results

**Fig. 1:**
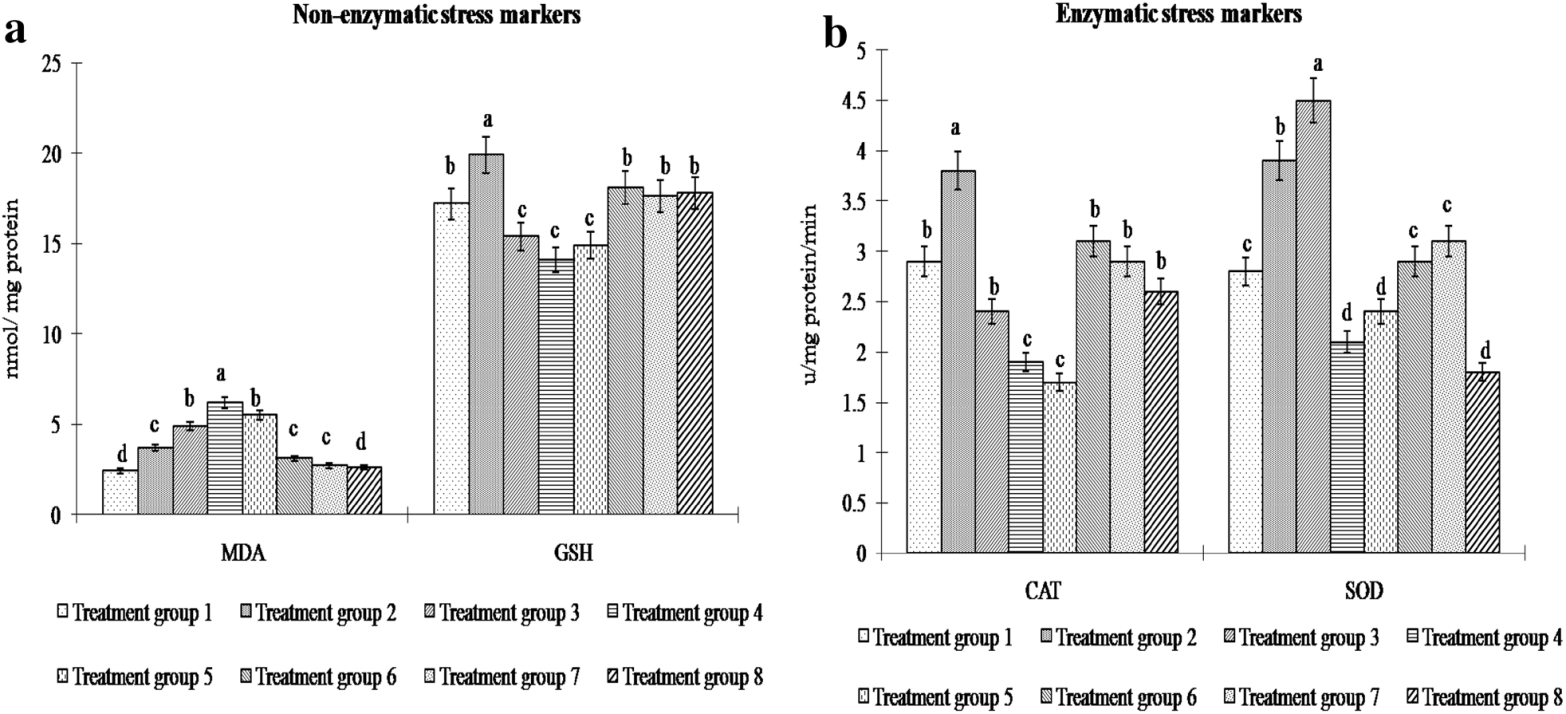
Activity of **a** non-enzymatic [MDA, GSH] and **b** enzymatic [CAT, SOD] antioxidants in hepatocytes of *Labeo rohita* in control [Treatment group 1], H_2_O_2_ (12.5 µM) [Treatment group 2], H_2_O_2_ (25 µM) [Treatment group 3], H_2_O_2_ (50 µM) [Treatment group 4], H_2_O_2_ (50 µM) + melatonin (25 μg/ml) [Treatment group 5], H_2_O_2_ (50 µM) + melatonin (50 μg/ml) [Treatment group 6], H_2_O_2_ (50 µM) + melatonin (100 μg/ml) [Treatment group 7], melatonin (100 μg/ml) [Treatment group 8]. Notes: *MDA* malonaldialdehyde, *GSH* reduced glutathione, *CAT* catalase, *SOD* superoxide dismutase. Data are means ± SE; (n = 6). Different alphabets indicate significant difference (P < 0.05) in mean values of corresponding parameter between different treatment groups. Treatment group with one particular letter (such as ‘d’) has significantly different mean value (P < 0.05) of the corresponding parameter than that in other treatment groups that show different letter (such as either ‘c’, ‘b’ or ‘a’)

Malondialdehyde level (MDA) was noted to increase significantly (P < 0.05) in all H_2_O_2_-challenged groups (Treatment groups 2–4) in a dose dependent manner compared to that in control (Treatment group 1). Level of non-enzyme antioxidant GSH showed a significant (P < 0.05) increase after H_2_O_2_ treatment at the concentration of 12.5 µM (Treatment group 2) compared to that in control hepatocytes (Treatment group 1), but it decreased significantly (P < 0.05) after H_2_O_2_ treatment at 25 µM and 50 µM concentrations (Treatment groups 3, 4) (Fig. 1a). Level of the antioxidant enzyme CAT increased significantly (P < 0.05) in treatment group 2 compared to that in treatment group 1. However, it showed a significant decrease (P < 0.05) following H_2_O_2_ treatment at higher concentrations (Treatment groups 3, 4). Activity of the other antioxidant enzyme, SOD showed a significant (P < 0.05) increase in treatment groups 2 and 3, followed by a sharp decrease in treatment group 4 (Fig. 1b). Melatonin treatment on the highest dose of H_2_O_2_-challenged group (50 µM) significantly (P < 0.05) reduced the MDA level, and increased the GSH level close to those in control hepatocytes (Fig. 1a). Activity of SOD and CAT was significantly (P < 0.05) elevated to the basal level in treatment groups 6 and 7 (Fig. 1b). Treatment with only melatonin (Treatment group 8) exhibited no significant difference (P > 0.05) in MDA, GSH and CAT levels compared to those in control hepatocytes (Treatment group 1) (Fig. 1).

**Fig. 2:**
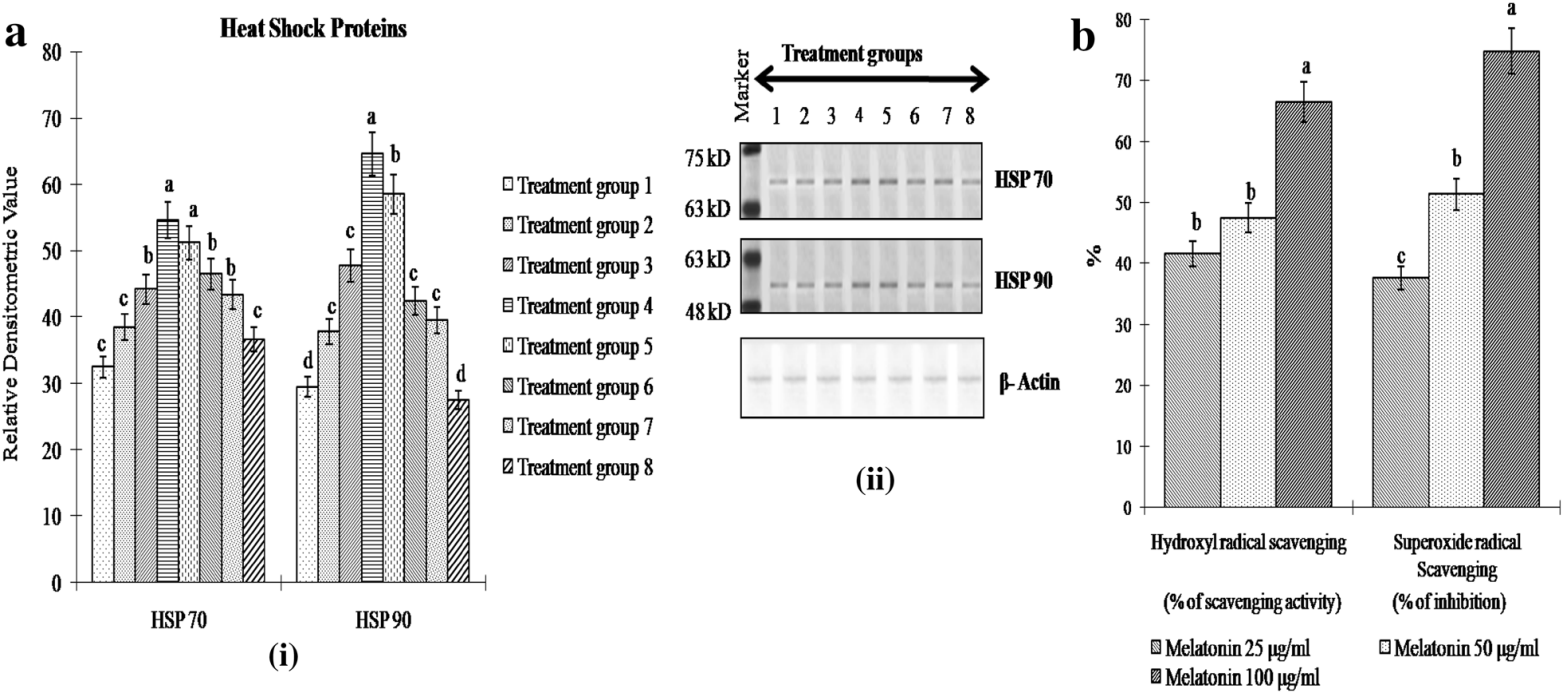
**a** Heat Shock Protein (HSP70 and HSP90) expression level in hepatocytes of *Labeo rohita* in control [Treatment group 1], H_2_O_2_ (12.5 µM) [Treatment group 2], H_2_O_2_ (25 µM) [Treatment group 3], H_2_O_2_ (50 µM) [Treatment group 4], H_2_O_2_ (50 µM) + melatonin (25 μg/ml) [Treatment group 5], H_2_O_2_ (50 µM) + melatonin (50 μg/ml) [Treatment group 6], H_2_O_2_ (50 µM) + melatonin (100 μg/ml) [Treatment group 7], melatonin (100 μg/ml) [Treatment group 8]. Panel on right side depicts representative bands for corresponding treatment groups. β-actin used as internal control. **b** Hydroxyl and Superoxide radical scavenging activity of melatonin at three different concentrations (25, 50 and 100 μg/ml) in hepatocyte of *Labeo rohita*. Notes: *HSP* heat shock protein. Data are means ± SE; (n = 6). Different alphabets indicate significant difference (P < 0.05) in mean values of corresponding parameter between different treatment groups. Treatment group with one particular letter (such as ‘d’) has significantly different mean value (P < 0.05) of the corresponding parameter than that in other treatment groups that show different letter (such as either ‘c’, ‘b’ or ‘a’)

H_2_O_2_ treatment increased both HSP70 and HSP90 expression level in a dose dependent manner. Treatment group 4 showed the highest HSP70 and HSP90 expression level and those were significantly higher (P < 0.05) compared to treatment groups 1, 2 and 3 (Fig. 2a). Both HSP70 and HSP90 expression level was observed to decrease gradually with the increasing concentrations of melatonin treatment, and treatment group 7 showed significantly lower (P < 0.05) HSP70 and HSP90 expression level compared to those in treatment group 4. Treatment with only melatonin (Treatment group 8) exhibited no significant difference in HSP70 and HSP90 expression level compared to those in control (Treatment group 1) (Fig. 2a). Melatonin at 100 µg/ml concentration was found to exert the highest hydroxyl and superoxide scavenging activity (Fig. 2b).

**Fig. 3:**
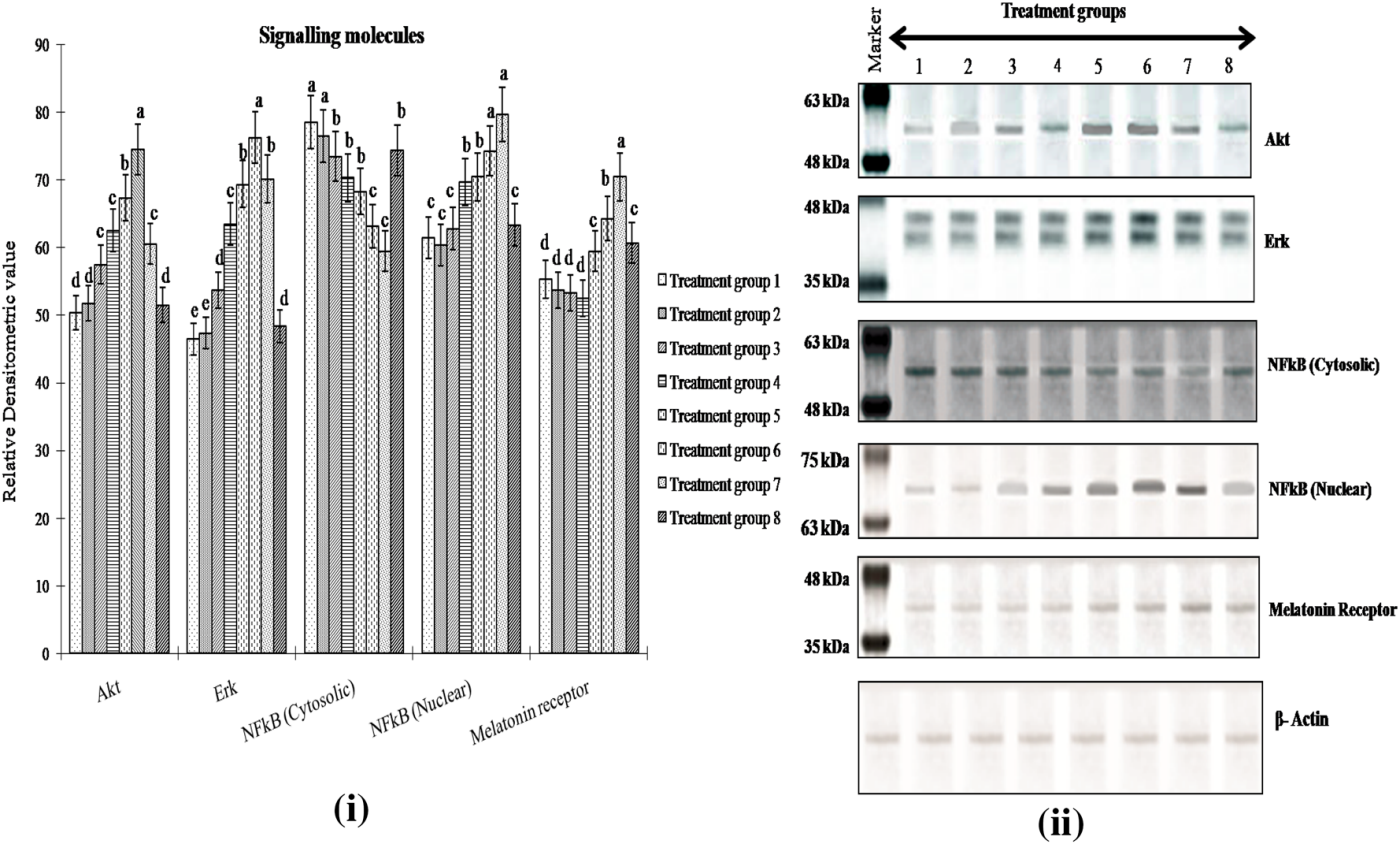
Expression level of Akt, Erk, NFkB (cytosolic), NFkB (nuclear) and melatonin receptor protein (MTR) in hepatocytes of *Labeo rohita* in control [Treatment group 1], H_2_O_2_ (12.5 µM) [Treatment group 2], H_2_O_2_ (25 µM) [Treatment group 3], H_2_O_2_ (50 µM) [Treatment group 4], H_2_O_2_ (50 µM) + melatonin (25 μg/ml) [Treatment group 5], H_2_O_2_ (50 µM) + melatonin (50 μg/ml) [Treatment group 6], H_2_O_2_ (50 µM) + melatonin (100 μg/ml) [Treatment group 7], melatonin (100 μg/ml) [Treatment group 8]. Panel on right side depicts representative bands for corresponding treatment groups. β-actin used as internal control. Notes: *Akt* protein kinase B, *Erk* extracellular signal regulated kinase, *NFkB* nuclear factor kappa B, *MTR* melatonin receptor. Akt, Erk and MTR measured only in cytosolic fraction. Data are means ± SE; (n = 6). Different alphabets indicate significant difference (P < 0.05) in mean values of corresponding parameter between different treatment groups. Treatment group with one particular letter (such as ‘d’) has significantly different mean value (P < 0.05) of the corresponding parameter than that in other treatment groups that show different letter (such as either ‘c’, ‘b’ or ‘a’)

Expression level of Akt and Erk1/2 in hepatocytes of *Labeo rohita* increased with increase in concentrations of H_2_O_2_ treatment, and treatment group 4 showed significantly higher (P < 0.05) expression level of Akt and Erk1/2 compared to treatment group 1. Akt and Erk1/2 protein expression level continued to increase significantly (P < 0.05) in treatment groups 5 and 6, but reduced again in treatment group 7. No significant alteration was observed in Akt protein expression following administration of only melatonin than that in control hepatocytes (Fig. 3). Treatment group 1 showed the highest expression level for cytosolic NFkB protein, and it decreased gradually from treatment group 2 to treatment group 7 (Fig. 3). On the other hand, significant increase (P < 0.05) of NFkB protein in the nuclear fragment was noted following H_2_O_2_- and melatonin-treated hepatocytes in a dose-dependent manner. Melatonin receptor protein (MTR) expression was observed to increase significantly (P < 0.05) in a dose dependent manner after melatonin treatment than all other groups and treatment group 7 showed the highest MTR expression level. MTR expression level significantly increased (P < 0.05) when treated with only melatonin than that in the control group (Fig. 3).

**Fig. 4:**
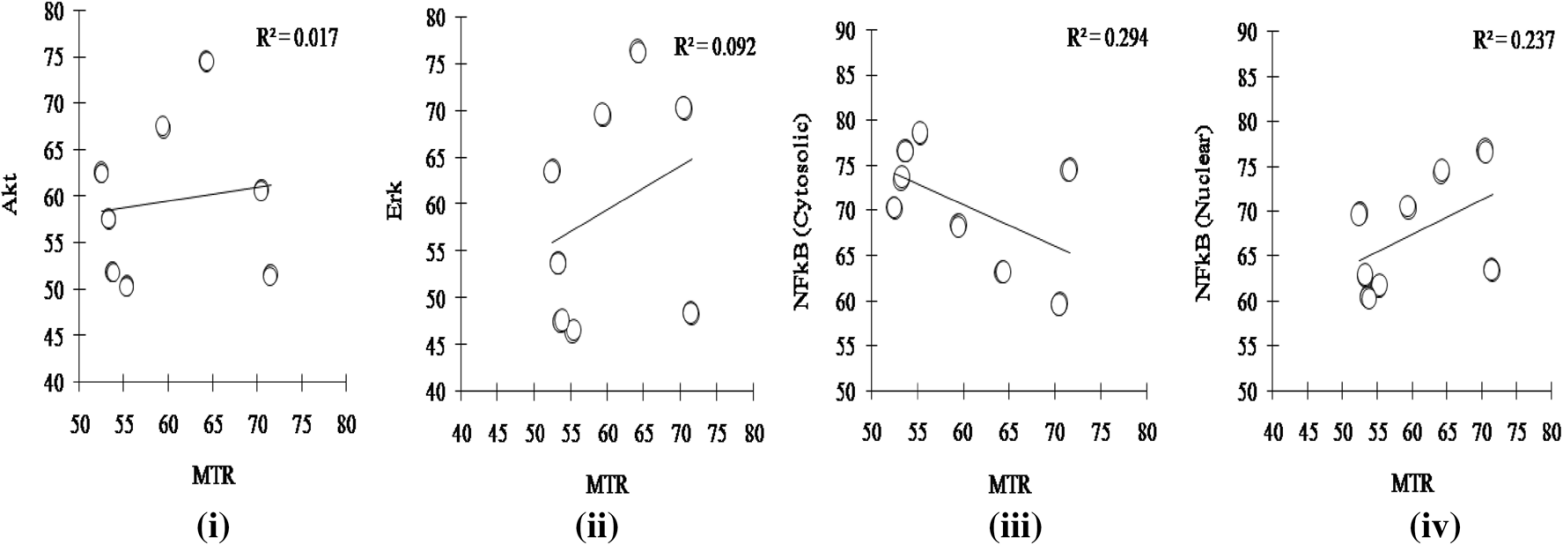
Scatter plots representing single linear regression analysis of different signaling molecules against Melatonin receptor protein. R^2^ denotes goodness of fit (P < 0.05). Notes: *RDV* relative densitometric value, *Akt* protein kinase B, *Erk* extracellular signal regulated kinase, *NFkB* nuclear factor kappa B, *MTR* melatonin receptor

Correlating the values of MTR with NFkB (cytosolic and nuclear fractions), MAPK and Erk through linear regression analysis (Fig. 4), it was observed that MTR expression in the hepatocytes is comparatively more correlated with the level of cytosolic and nuclear NFkB (R^2^ = 0.294 and 0.237, respectively) rather than with Erk (R^2^ = 0.092) or Akt (R^2^ = 0.017).

## Discussion

Oxidative stress due to accumulation of free radical and reactive oxygen species constitutes a major threat to the organism. Cellular response towards such stress is an intricate phenomenon that involves proper harmonization of multiple signaling pathways that act in concert to maintain natural cellular activity and tissue functions. Oxidative damage and associated adaptive mechanisms in cells include the induction of a subset of stress proteins (HSP), activation of kinases (MAPK), and modulation of transcription factors like NRF2 and/or NFkB.

High MDA level denoted an elevated oxidative stress [20] as noted in H_2_O_2_-treated fish hepatocytes here. A significant reduction in the level of MDA and the modulation of enzymatic (SOD, CAT) as well as non-enzymatic (GSH) antioxidants after melatonin treatment of H_2_O_2_-stressed fish hepatocytes (Fig. 1) collectively demonstrated antioxidative actions of melatonin in hepatocytes of the concerned fish. SOD catalyzes the dismutation of O_2_^·−^ to O_2_ and H_2_O_2_, and represents the first line of enzymatic defense against ROS. Thus, in the present study, with increased stress level at the beginning, SOD increased till 25 µM dose of H_2_O_2_. CAT catalyzes the decomposition of H_2_O_2_ to produce water and molecular oxygen; it also plays a major role in protecting cells against further oxidative damage. In the current study the application of 25 µm dose of exogenous H_2_O_2_ cumulatively crosses the tolerable limit of cellular H_2_O_2_ which immediately suppressed the normal CAT activity. Therefore, CAT activity gets hampered during the initial phase of H_2_O_2_ treatment prior to SOD. In contrast, SOD decreases at the later stage of H_2_O_2_ treatment, when highest dose of H_2_O_2_ accumulates excess ROS and free radicals and damages the hepatocytes further. Similar response of SOD-CAT system was noted in an earlier work of [21] in the mammalian hepatocytes. Present study by showing a significant alteration in the activity of both GSH and antioxidant enzymes (SOD-CAT system) after melatonin treatment of isolated carp hepatocytes provides persuasive evidence of an important role of the glutathione system as well as SOD-CAT system as the major defense against oxidative stress during H_2_O_2_-induced oxidative damage.

The cellular response to stresses is represented at the molecular level by rapid synthesis of molecular chaperones such as HSP70 and HSP90 [22]. Through the chaperone function, HSPs are likely to protect proteins against denaturation or oxidative inactivation, or assist in the folding of stress-modified proteins [23]. Both HSP70 and HSP90 chaperones cooperate in client protein folding to an active conformation, protein stabilization, and protein turnover by the formation of multichaperone complexes [24]. The recruitment and regulation of Hsp90 client proteins is connected to the function of Hsp70 and a multitude of other cochaperones. HSP90 initially tries to buffer the effect of oxidative damage followed by Hsp70-dependent protective events [24]. Similar pattern of HSP90 and HSP70 expression level in H_2_O_2_-stressed fish hepatocytes was observed in the present study. Significant reduction (P < 0.05) in HSP90 and HSP70 expression after melatonin treatment of H_2_O_2_-exposed hepatocytes indicated the protective efficacy of melatonin against oxidative stress (Fig. 2a).

HSPs have the inherent ability to decrease the intracellular level of ROS by modulating metabolism of glutathione to maintain it in a reduced state [25]. In the present study as well, melatonin treatment of H_2_O_2_-stressed fish hepatocytes restored GSH levels similar to that in control hepatocytes (Fig. 1). HSP may also act to promote survival and prevent cell death through the modulation of other important signaling pathways affected by excessive ROS accumulations as observed in the fish hepatocytes in the current study. HSP has been reported to regulate ERK1/2 in the MEK-ERK pathway [26]. Akt interact with HSP to perform different processes in cellular signaling mechanism [27–29]. Therefore, activation of ERK1/2 and Akt expression after peroxide treatment support previous observation regarding HSP90 upregulation in the pollutant-stressed fish hepatocytes [13]. Melatonin treatment at highest dose was quite eminent to regulate the MAPK pathway by decreasing the expression of both ERK1/2 and Akt to basal level. Earlier report suggests that Akt activation is analogous to HSP induction. Formation of Akt-HSP complex stabilizes Akt that protects the cells from undergoing apoptosis [27]. A direct correlation between resistance to oxidative stress and activation of ERK1/2 or Akt has also been put forth earlier [30]. Thus ERK1/2 and Akt induction in H_2_O_2_-stressed hepatocytes as compared to their control counterparts reinforce the view that ERK and PI3 K/Akt signaling pathways contribute to the survival of cells under H_2_O_2_ related stress as reported earlier [31, 32].

The NFkB family of transcription factors has a fundamental role in protecting the cells from apoptosis during exposure to a variety of stressors [33, 34]. Expression pattern of cytosolic and nuclear NFkB were analyzed to establish their influence on survival and health of H_2_O_2_-stressed hepatocytes together with signaling modulators ERK1/2, Akt in the hepatocyte. Significant activation of ERK1/2 and Akt and an enhanced nuclear NFkB expression was noted in H_2_O_2_-stressed fish hepatocytes. In these hepatocytes as compared to control counterparts, NFkB levels significantly increased in the nucleus indicating the movement of activated NFkB from cytosol following peroxide stress. Under normal condition, NFkB is present in the cytoplasm in an inactive state and translocates from cytosol to nucleus upon activation by stressor stimuli [35]. During H_2_O_2_-related oxidative stress, enhanced activation of NFkB characterized by its translocation from cytosol to nucleus was evident which suggests the prevalence of pro-survival mechanism mediated by NFkB in stressed fish hepatocytes. Such finding was similar to those reported in hyperoxia model of oxidative stress, where significant increase in NFkB activation and translocation in the nucleus has been demonstrated [13]. In an earlier study, altered ERK expression was noted in response to oxidative stress in fish hepatocytes [36]. Since high dose of H_2_O_2_ treatments lead to excessive ROS production, it activates the transcription factor to modulate the antioxidant system. A few earlier reports demonstrated that activation of NFkB in response to hydrogen peroxide is cell type-specific and an increase in ROS may result in NFkB activation [37]. In certain cell types, oxidative stress is a potent activator of NFkB and other transcription factor such as MAPK which can have important consequences for cell survival and cell signalling. In our current study once ROS levels increased at higher H_2_O_2_ treatment it affects the antioxidant status specifically glutathione metabolism that causes increased lipid peroxidation (Fig. 1a). In such circumstances, HSPs act as the central mediators to regulate the NFkB activation and translocation process which in turn modulate the ERK/Akt signaling cascade. Current study provides strong support to earlier hypothesis regarding dose dependent effect of melatonin on induction of cellular signaling process under severe oxidative damage. Only the highest dose (100 µg/ml) of melatonin treatment in H_2_O_2_-stressed fish hepatocytes could reduce expression level of ERK and Akt (Fig. 3) indicating that this particular dose would be effective to ameliorate H_2_O_2_ induced oxidative stress in fish hepatocytes.

Several mechanisms of action have been proposed to explain the protective role of melatonin against different stress conditions. Among them, the most significant is the modulation of signaling transduction pathways triggered by melatonin through its receptor protein. Involvement of melatonin receptors (MTR) in melatonin-induced activation of the ERK/Akt signaling pathway is confirmed in the present study. The expression of melatonin receptor protein (MTR) in the isolated fish hepatocytes is indicative to its possible role in mediating melatonin actions on the antioxidative system. Increase in MTR expression with subsequent induction of ERK/Akt transcription factor essentially lend support to the idea that the stimulatory influence of melatonin on isolated hepatocytes in H_2_O_2_-treated carp could be mostly due to its specific receptor mediated actions on different antioxidative enzymes [38]. Akt modulates NFkB, thus resulting in NFkB mediated transcription of prosurvival and antioxidative genes to maintain cellular health [39]. Correlation results are also indicative of melatonin regulated NFkB activation through the induction of its receptor protein (Fig. 4).

Melatonin was also noted to successfully control the superoxide and peroxide radical by its direct scavenging characteristic (Fig. 2b). Melatonin employs antioxidant effects by controlling the production of ROS, restraining the intracellular free radical stress and therefore determines the development of stress recovery. In the current study we tried to demonstrate that melatonin, through its role as a direct scavenger of radical oxygen and also through activation of antioxidant enzymes, can effectively protect the hepatocytes against oxidative stress and further inflammatory damage. The protective role of melatonin as an antioxidant agent has been characterized in detail in other cell types [40]. Thus, in fish hepatocytes, H_2_O_2_-induced modulation of the extracellular signal-regulated protein kinases (ERK and Akt), and some of their regulatory protein effectors (HSPs and NFkB) were strongly regulated by melatonin, as well as H_2_O_2_-induced activation of antioxidant status as reported by some earlier report [40].

Treatment of fish hepatocytes only with melatonin (Treatment group 8) showed little variations in different parameters compared to those in control hepatocytes (Treatment group 1). Moreover, this group showed significantly lower MTR expression level compared to treatment group 7. Collectively, the results from the present study indicate that melatonin might exert its anti-stress effect by melatonin receptor mediated action on the antioxidative enzymes and modulation of the Akt/ERK/NFkB system only after pre-exposure to H_2_O_2_-induced oxidative stress.

## Conclusions

Current study describes the effect of melatonin on H_2_O_2_-induced stress response and heat shock protein expression in carp hepatocytes, focusing on modulation of NFkB/Akt/ERK signal transduction pathway. The result of this study indicates that melatonin treatment of H_2_O_2_-stressed fish hepatocytes may restore basal cellular oxidative status in a dose dependent manner. Treatment with melatonin at the concentration of 100 µg/ml is required to combat accumulation of free radical and reactive oxygen species, and ameliorate the antioxidant status through modulation of different transcription factors and molecular chaperons. The interrelation between the induction of HSPs and antioxidant factors in the fish hepatocytes and subsequent effect on signaling cascade in response to ROS accumulation and free radical damage is also highlighted. The study illustrates how peroxide accumulation in the hepatocytes and ensuing oxidative stress affect the Akt/ERK signal. The possible regulatory factor might be HSP that acts as the client protein to correlate the antioxidant status and transcription regulation.

Present experimental outcome directly addresses mechanistic pathways to advance our understanding regarding role of melatonin to combat superoxide attack and free radical induced damage in fish hepatocytes. The results of our study also argue in favor of a critical role of melatonin receptors on the hepatocytes in mediating stimulatory actions on the synthesis and/or activity of several antioxidative agents. Therefore it can be hypothesized that receptor mediated action of melatonin organize the expression of different transcription factors in the stressed hepatocytes which are known to play regulatory role in de novo synthesis of different antioxidative enzymes. Moreover, in light of our findings it can be hypothesized that melatonin may also be effective during liver dysfunction due to severe oxidative damage. Nonetheless, considering limitations of current study, probable effect of higher doses of melatonin in amelioration of oxidative stress and possible actions of melatonin on other MAPK remain unresolved and thereby warrant further study.
